# A qualitative exploration of chronic pain management of older adults in remote and rural settings

**DOI:** 10.1007/s11096-023-01607-8

**Published:** 2023-07-01

**Authors:** Tesnime Jebara, Elaine Youngson, Natalie Drummond, Gordon Rushworth, Sharon Pfleger, Ian Rudd, John MacLeod, Martin Wilson, Nicola Bailey, Scott Cunningham

**Affiliations:** 1 King’s College London, London, SE58AF UK; 2 Robert Gordon University, Aberdeen, AB107GJ UK; 3 NHS Grampian, Aberdeen, UK; 4 NHS Highland, Inverness, UK

**Keywords:** Aged, Ageing, Chronic pain, Population health management

## Abstract

**Background:**

The World Health Organization predicts that the number of older adults will nearly double between 2015 and 2050. Older adults are at a higher risk of developing medical conditions such as chronic pain. However, there is little information about chronic pain and its management in older adults especially those residing in remote and rural areas.

**Aim:**

To explore views, experiences, and behavioural determinants of older adults regarding chronic pain management in remote and rural settings in Scottish Highlands.

**Method:**

Qualitative one-to-one telephone interviews were conducted with older adults with chronic pain residing in remote and rural areas in the Scottish Highlands. The interview schedule was developed by the researchers, validated, and piloted prior to use. All interviews were audio-recorded, transcribed, and independently thematically-analysed by two researchers. Interviews continued until data saturation.

**Results:**

Fourteen interviews were conducted with three key themes emerging: views and experiences with chronic pain, need to enhance pain management, and perceived barriers to pain management. Overall, pain was reported as severe and negatively impacted lives. Majority of interviewees used medicines for pain relief but noted that their pain was still poorly controlled. Interviewees had limited expectation for improvement since they considered their condition a normal consequence of ageing. Residing in remote and rural areas was perceived to complicate access to services with many having to travel long distances to see a health professional.

**Conclusion:**

Chronic pain management in remote and rural areas remains a significant issue among older adults interviewed. Thus, there is a need to develop approaches to improve access to related information and services.

## Impact statements

- This work confirms that the management of chronic pain remains a challenge in older adults in remote and rural settings.
- Participants indicated that there is a need to improve access to chronic pain management services because this was seen as a significant barrier to effective pain control.
- Service redesign that integrates triage is needed to ensure complex cases are referred promptly to specialist pain management services and less complex cases are managed by practitioners in primary care.
- A key facet of service redesign should include consideration of synergistic utilisation of all the members of the multidisciplinary team.

## Introduction

The World Health Organization (WHO) has predicted that the world's population of over 60 years is expected to nearly double from 12 to 22% between 2015 and 2050 [1], rising from 900 million to 2 billion by 2050 [2]. The WHO has a vision for a world in which everyone can live a long and healthy life. However, ageing is a challenge to many healthcare systems as it is associated with multiple common conditions and complex health states. Chronic pain associated with some of these medical conditions can lead to older adults experiencing a lower quality of life [2].

Chronic pain is defined as pain which has been present for 12 weeks or more [3]. A survey conducted across Europe estimated that almost one fifth of the adult population are affected by moderate to severe chronic pain [4]. An updated study examining recent trends in pain prevalence among adults aged 50 and above across Europe in the period between 2004 and 2015 showed that population-level pain prevalence ranges from about 30% to 60% depending on the country and year [5]. Pain has a large impact on people’s general wellbeing, quality of life, and ability to function and is often linked to sleep disturbances, depression, as well as consuming vast healthcare resources [6]. Older adults are particularly affected, with pain estimated to double after the age of 60 years and to increase every 10 years thereafter [7]. A systematic review of chronic pain prevalence in the UK showed that chronic pain prevalence increased steadily with age [8].

In Scotland, there is a move towards improving care of older adults especially in terms of chronic pain management as articulated in the Scottish Government’s ‘2020 Vision for Health and Social Care’ [9], “Getting to GRIPS with chronic pain” [10], and the ‘Quality prescribing for chronic pain: A guide for improvement 2018–2021’ [11]. As a result, patients must be appropriately assessed, offered an integrated person-centred plan of treatment, and reviewed regularly.

Scottish Intercollegiate Guideline Network (SIGN) 136 focuses on the management of chronic pain highlighting the importance of assessment, supported self-management and therapies [12]. Significant emphasis is placed on pharmacological therapies of non-opioid analgesics, opioids, anti-epilepsy medicines, anti-depressants, and combination therapies. Key roles of psychological therapies, physical therapies and complementary therapies are also emphasised [12]. Key issues for older adults include the potential for adverse drug reactions, interactions with other concomitant medicines being taken, adherence and ability to access and use some of the recommended therapies [13].

Management of medical conditions in remote and rural areas is a complex issue facing many countries internationally due to inequalities in healthcare including lack of access, poor quality of services, and workforce issues. The Australian Institute of Health and Welfare reported that, those residing in remote and rural areas of Australia, on average, have shorter lives and higher levels of disease and injury compared to those residing in metropolitan areas. They also report barriers to receiving healthcare including no access to general practitioners (GP) or specialists nearby due to health workforce shortages in these locations [14]. Similarly, geographic variability in healthcare policy, access, and utilisation were observed in Canada leading to poorer outcomes in remote communities despite Canada’s publicly funded healthcare system [15]. A cross-sectional study evaluating access to health care services along the rural–urban continuum in Canada reported lower usage of specialist physicians and poorer health status in those residing in rural areas compared to major urban centres [16].

While NHS Highland is the largest geographical health authority in the United Kingdom, only 31.3% of the Highland Council population live in ‘urban areas’ (defined as settlements ≥ 10,000 people) compared to 70.8% of the entire population of Scotland. Moreover, within Highland, 37.9% of the population live in ‘remote rural’ locations (defined as settlements with a population of less than 3,000 people, and with a drive time of over 30 min to a settlement of 10,000 or more) [17]. Given the challenges in accessing healthcare generally in remote and rural areas, patients with chronic pain are likely to face difficulties in controlling their pain. However, there are limited published studies exploring chronic pain management in these areas, especially in older adults.

A recent survey study was conducted in the Scottish Highlands to describe the perspectives of older adults on their chronic pain management. Chronic pain was reported by a quarter of survey respondents, and it often impacted many aspects of daily living and physical function leading to negative psychological consequences (e.g. kinesiophobia, sadness). Those experiencing chronic pain were significantly higher users of healthcare resources and living in more deprived areas. Pain was most commonly managed by a GP using paracetamol alone or in combination with opioids and other analgesics, with few respondents reporting using non-pharmaceutical therapies [18].

### Aim

This research aimed to continue the work reported in the previous survey study and explore and understand the views, experiences, and behavioural determinants of older adults regarding chronic pain management in remote and rural community settings in the Scottish Highlands.

### Ethics approval

This research was reviewed by the Ethics Review Panel, School of Pharmacy and Life Sciences, Robert Gordon University on 15/08/2019 (Ref S188). The advice of the North of Scotland Research Ethics Committee and NHS Highland Research and Development Management Committee has been sought and NHS approval was not required. Signed, informed consent was obtained from all research subjects via mail prior to conducting any field work.

## Method

### Study design

The research methodology was undertaken according to an interpretivism philosophy using a qualitative research methodology. Individual semi-structured telephone interviews were conducted with older adults within remote and rural geographic locations as this was considered the most appropriate method to facilitate in-depth rich data capture and analysis.

### Sampling and sampling approach

Older adults (defined as those aged 75 years and over) living within remote and rural areas of NHS Highland who are experiencing chronic pain (defined as pain lasting more than 12 weeks) who participated in the previous survey-based research project [18] and indicated on the questionnaire returned that they were willing to take part in further research were invited to participate in the interviews. Those willing were mailed the participant information sheet and asked to complete a consent form and provide details of the best days and times of the week for the interview. All respondents who agreed to participate and fitted the inclusion criteria were interviewed.

### Development of interview schedule

The semi-structured interview schedule was developed based on the aim of this study, the published literature on the topic, and The Theoretical Domains Framework (TDF). The TDF summarises key elements of 33 theories and proposes that determinants of behaviour cluster into 14 domains [19]. Those domains most relevant (e.g. knowledge, beliefs about capabilities and consequences, motivation and goals, environmental context and resources) were used to guide construction of interview core questions. Furthermore, the content of relevant literature such as the ‘Scottish Intercollegiate Guidelines Network on Management of chronic pain’ [12] and the AGS Panel on persistent pain in older persons: The management of persistent pain in older persons [7] guided construction of the interview schedule. Credibility of the interview schedule was enhanced through review by key national expert researchers and practitioners and was piloted with two participants. The key sections and questions of the interview schedule are presented in Box 1.

**Box 1:** Semi-structured interview schedule

*Section 1. Your pain* Are you currently experiencing any chronic pain (lasting more than 12 weeks)? On a scale of 0 to 10 (with 10 being the worst pain) can you tell me how severe your pain has been over the past week? Can you please describe the main site(s) of your pain and its nature (sharp, shooting, stabbing etc.)? Can you outline how this has affected you e.g. physical function, daily activities, hobbies etc.? *Section 2 . Who helps with your pain?* Which healthcare professionals do you speak to regarding your pain? Why? How often? Out of these, can you tell me who you mainly speak to about your pain and why? Have you been referred to a specialist pain clinic? If so what was your experience of this? *Section 3. How do you manage your pain?* How do you manage your pain?– consider medicine (prescribed, over the counter, herbal) and non-medicine (acupuncture, physiotherapy, TENS) How often do you use these?- regular or occasional use How many medicines are you on for pain and can you tell me what they are? How do you find taking your medicines for pain? On a scale of 0–10 (10 being most effective), how effective do you find your medicines for pain? What about side effects, are you experiencing any? Do these trouble you at all? What about non-medicine methods—do you find these effective? *Section 4. How could your pain management by improved?* Since you are living in a remote and rural area, do you have any difficulties in accessing chronic pain management regimens or any of its associated information and support? Do you expect your health to improve if you continue to use these management regimens as recommended? Why/why not? Are there any changes you would like to be made to your current management to improve your chronic pain control? Why do you feel this way? Is there any way in which the pharmacy services could be improved for you?	

### Data generation

All interviews were conducted by telephone from November 2019 to January 2020 by undergraduate students as part of their final year project. All interviewers received training on qualitative research and how to conduct interviews prior to starting data generation. Interviews continued until the point at which saturation of themes was deemed to have occurred [20]. Following the 10th interview, additional 4 interviews were conducted to test if any additional data emerged. Following the 14th interview, it was determined that data saturation was reached. All interviews were audio recorded, transcribed verbatim, and checked for reliability of transcribing prior to analysis.

### Data analysis

Thematic analysis of the data was undertaken using the TDF and the interview schedule as thematic guides. Analysis was performed by two research members independently with any disagreements resolved through discussion. In reporting this study, the Consolidated Criteria for Reporting Qualitative Studies (COREQ) were followed [21].

### Research trustworthiness

Several steps were taken to enhance the trustworthiness of the research data and findings and reduce risk of bias as outlined by Hannes [22]. First, to ensure credibility, the data generation tool was carefully developed and piloted prior to use, all interviews were audio-recorded and transcribed verbatim, and data was independently analysed and presented in the form of themes supported by verbatim quotes. The research methods including participants’ inclusion criteria and the process of data generation were clearly outlined to improve transferability of research findings. All transcripts were transcribed, checked, and analysed by at least two research team members to enhance the research’s dependability. Confirmability was considered through the analysis being grounded in the data and the clear outline of research team’s roles throughout the study process.

## Results

From the previous survey study, 120 participants agreed to take part in the follow-up qualitative interviews. However, only 14 met the inclusion criteria and were subsequently invited to take part (Table 1). Half of the interviewees were female, lived alone, held a college degree, and resided in a very remote rural area (class 8). On average, the interviews were 30 min in duration with three key themes emerging: description of chronic pain, management of pain, and barriers to pain management.

**Table 1:** Interviewees demographics

Participant code	Deprivation category	Urban and rural classification*	Gender	Age	Living alone	Ethnicity	Education level
N1	4	2	Female	75–79	Missing	White	School
N2	2	8	Male	80–84	Yes	White	College
N3	2	8	missing	85–89	No	White	College
N4	3	8	missing	90 or over	Missing	White	College
N5	4	5	Female	75–79	Yes	White	School
N6	5	2	missing	75–79	No	White	School
N7	3	8	Male	75–79	Missing	White	School
N8	5	5	Male	90 or over	Yes	White	College
N9	3	4	Female	75–79	Yes	White	College
N10	2	4	Female	80–84	Yes	White	School
N11	4	8	Female	80–84	No	White	College
N12	4	8	Female	80–84	Yes	White	College
N13	4	8	Male	75–79	Missing	White	University
N14	4	7	Female	80–84	Yes	White	School

*Based on Scottish Government Urban Rural Classification [17]

### Views and experiences with chronic pain

All interviewees reported experiencing chronic pain ranging in nature from “*dull”* to “*sharp*” and “*stabbing*” (Table 2). The most common cause of pain was arthritis with cold weather being the main trigger. Pain was often severe with the majority rating it a 7 on a scale of 0 (no pain) to 10 (most severe pain they ever experienced). Most experienced pain for a prolonged period of time ranging from 4 months to 24 years.

**Table 2:** Description of pain

	Location	Severity*	Causes/triggers	Duration
N1	Knee, back	7	Polymyalgia	Long time
N2	Wrist	7	Weather	1 year
N3	Lower spine	4	Accident	13 years
N4	Hips to toes	8	Arthritis	4 months
N5	Keens, legs, back	9	Accident	Number of years
N6	Hip	7	Arthritis	Many years
N7	Hip down to leg	7	Metastasised cancer	15 years
N8	Knees, feet	2–7	Old age	Last few years
N9	Legs, hands, feet	7	Arthritis, weather	11 years
N10	Knees, hands, elbows	7	Arthritis	20 years
N11	Knees, ankles, hip, shoulder	2	Weather	24 years
N12	Knuckles, hands, hip	7 or 8	Arthritis	2 years
N13	Hip down to leg	6 or 7	Arthritis	1.5 years
N14	Legs, feet	5	Arthritis	Long time

*Severity scale of 0–0 (with 10 being the worst pain)

Chronic pain negatively impacted interviewees’ daily activities including their ability to exercise, do chores around the house, or even walk.

“*When I get up in the morning I can hardly walk because of the pain in my toes*.” N4

“*I can’t walk far, and I can’t do things in the house that I would normally do*.” N10

### Need to enhance pain management

Almost all interviewees “*would go to [their] doctor”* (N12) for any pain-related queries or flare-ups with very few aware of or referred to a specialist pain clinic.

*“Pain clinic? No, I didn’t even know they existed!”* N13

They used different management approaches to control their pain with varying effectiveness (Table 3) but almost all relied mainly on pharmacological options to ease their pain. However, they reported frustration with their chronic pain management which was often associated with uncomfortable side effects.

“*Originally, I was on diclofenac but after about 12 years, it interfered with my stomach… they put me onto Celebrex [celecoxib]… and then the same thing happened again… I have spent hundreds of pounds on different things but nothing has done any good*.” N11

“*When I started taking the gabapentin, I put on 2 and a half stone of weight. And because I put on the weight, I have now got diabetes and that makes me very breathless*.” N14

**Table 3:** Pain management modalities used by interviewees

	Pharmacological approaches			Non-pharmacological approaches		
	Name	Effectiveness (0 not effective—10 most effective)	Issues	Name	Effectiveness (0 not effective—10 most effective)	Issues
N1	Paracetamol	8	None	Exercise	8	None
N2	None	–	–	Physio	5	Not working
N3	Paracetamol Hydrocortisone injection	As needed 0	None	Physio and acupuncture	As needed	Access (150 miles away)
N4	Codeine and Anadin extra (aspirin, paracetamol, caffeine)	3	Drowsiness	None	–	-
N5	Diclofenac gel	10	None	Physio	Stopped	Not working
				Chiropractor	8	Cost
N6	Tramadol	8	None	None	–	–
N7	Oramorph, MST	8–9	Constipation, Access (MST)	Heat pad	2 (Stopped)	Not working
	Paracetamol	9	None			
	Pregabalin	8	None			
N8	Paracetamol	3	None	None	–	–
N9	Paracetamol	10	Nightmares	Heated blanket	9	None
N10	Cortisone injections	9 (Stopped due to cancer)	Deafness, cataracts	Physio, Acupuncture, heat pads	4–5 (Stopped)	Not working
	Co-dydramol	5	Constipation			
N11	Diclofenac, celecoxib	Stopped	Stomach pain	TENS machine	8–9	Burnt back
	Cortisone injections, paracetamol, Versatis [lidocaine] plasters, Ibuprofen gel	8–10	None	Acupuncture, physio	6–7	Cost and Access (11 miles away)
N12	Tramadol + paracetamol	7–8	None	Acupuncture	2–3 (Stopped)	Hot and burning
	Ibuprofen gel	6	None	Physio	8 (Stopped)	Access
				Massage	9	None
N13	Naproxen	6	None	Heat pad	2–3 (Stopped)	Not working
N14	Dihydrocodeine	Stopped	Not working	Physio	Stopped	None
	Gabapentin, duloxetine	7	Weight gain, Insomnia	Hot beanbag	–	–

One interviewee even reported self-managing their pain despite being satisfied with the care received for other conditions.

“*To be perfectly honest with you, in all other respects, I’ve had very good health care, now the question of pain management has hardly cropped up and I’m taking things into my own hands really, because I bought Naproxen from America*.” N13

Very few reported using non-pharmacological approaches to manage their pain successfully.

“*I got acupuncture for my back years ago, and I’ve had one or two acupunctures since then. And that kept it at a very good level. I haven’t had much bother with it really*.” N3

Some noted that the reason they do not seek medical help despite poor pain control is that health professionals tend to see pain as a natural consequence to ageing so there is less desire to address it.

“*I think that as you get older, people expect you to have arthritis and it’s just accepted that you have it. It’s not that they’re not interested in your pain, it’s just that it’s seen to be normal*.” N10

“*They sort of just ignored it in the beginning until it got really severe*.” N14

Overall, almost all interviewees accepted that their pain is related to their age and thus do not expect it to improve.

*“I don’t think I can [improve] now at my age, I don’t think there’s anything else they can do really… obviously I’ll never be able to get over it, but on the other hand I probably won’t die of it.”* N6

*“When you get to 95, nothing improves. You find that out eventually. You can’t beat old age.”* N8

But they were keen to receive more information on their pain and how they could manage it more effectively.

*“I’ve never asked for anything else, but I’ve never been offered anything else other than injections… maybe tell me how to manage it a bit better.”* N10

*“It certainly would be helpful to have specific advice on the various ways of managing pain… What you can do yourself, and what sort of medicinal treatment is available or might be available as well.”* N13

Generally, the majority of those interviewed were satisfied with the care they received and reported that, if they ever need help, they can rely on their providers for support.

*“The physio when it started, she gave me a set of exercises which were successful… It's up to me for the moment to resume the exercises and try to get rid of it myself. But I know, if I can’t, she’s there.”* N2

*“They come to me and see how I’m getting on; I have absolutely no complaints about that. They’re very good… they’ve kept me going for twenty years, and I’m not about to start complaining now.”* N7

### Perceived barriers to pain management

Living in remote and rural areas was considered a barrier to accessing the care needed to manage their pain as patients often had to travel for long distances for their appointments.

“*Our nearest hospital or anything like that is 150 miles away… It’s a 3 and a half hours or so drive to get across… There’s no [public] transport*… *By the time you got to Portree, the time you spend driving there and back, I think it’d knock out the good of the therapy*.” N3

“*I don’t have acupuncture or physio anymore… I used to go to aqua aerobics, which I really enjoyed but that’s in the next village and because I can no longer drive and the bus times aren’t suitable and nobody else from here goes to it from where I live so that’s ruled that out*.” N11

Moreover, they highlighted issues in securing appointments resulting in long waits to see someone regarding their pain.

“*If you could get an appointment with the doctor* [GP], *which you can’t get, it’s impossible. I was going on holiday just a few months ago and I was due to get my knee injections… [but]I couldn’t get an appointment*.” N5

*“I was meant to have an appointment [hospital outpatient] about six weeks ago but there’s no word at all… The issue is waiting for an appointment. I mean part of the problem is that they are short of staff now in the rheumatology department at XX hospital so I’m just left hanging really*.” N13

However, some were able to overcome this by relying on neighbours, a community car (for a reduced fare), or the patient ambulance service (a special ambulance provided by the NHS that transports patients to and from their appointments free of charge).

*“I’ve got very good neighbours I must admit there’s always somebody that’s willing to take me and if it comes to the push there’s a community car that take you out for a minimum price… when I have to go back to the hospital, I get the patient ambulance service which is absolutely brilliant thanks to the NHS.”* N12

Cost of certain non-pharmacological approaches that are not covered by the NHS was also highlighted as a potential barrier to effective pain control.

“*I don’t go often but I do when my back and leg are really bad… I stuck it out because it’s a lot of money to go to the chiropractor and we don’t get it free so I can’t go a lot*.” N5

“*I only had 3 sessions of it which you get on the national health… [Now] I have to actually pay to have to go and get a massage.*” N11

As a result, few noted that offering non-pharmacological treatments free of charge could help them control their pain without having to rely on medicines.

“*I would like the chiropractor to be on the national health. I think he’s worth his weight in gold because he knows every part of your body… I think if people could get the chiropractor on the NHS they wouldn’t need so many tablets.”* N5

## Discussion

### Summary of key findings

Fourteen interviews were conducted with three key themes emerging: views and experiences with chronic pain, need to enhance pain management, and perceived barriers to pain management. The majority attributed their pain to arthritis, scored it as a seven out of ten, and reported that it negatively impacted their daily activities but accepted it as a normal consequence to ageing. This view was also believed to be shared by healthcare professionals who were less likely to address chronic pain until it was severe. Almost all used pharmacological options to control it with very few using non-pharmacological approaches. Interviewees were satisfied with the care provided but expressed interest in receiving more information on their pain and how they can manage it better. Some noted difficulties in accessing pain management methods such as prolonged waiting times and having to travel long distances for appointments due to living in remote and rural areas. Thus, they noted a need to improve access to pain control approaches.

### Strengths and limitations

There is paucity of literature exploring chronic pain management especially amongst older adults residing in remote and rural areas. Thus, this study adds to the evidence base a useful insight into this topic. Using qualitative one-to-one interviews allowed in-depth exploration of interviewees’ views and experiences. The interview schedule was rigorously developed and piloted to ensure that all key aspects of the research were explored. To ensure confirmability and reduce bias, analysis was conducted by two researchers independently. Data were collected from the Scottish Highlands and thus might not be transferrable to other settings. Moreover, participants who expressed interest during a previous project were invited to be interviewed. Thus, it is likely that only views of those who are keen to participate or have strong opinions to share were explored so data must be interpreted with caution.

### Interpretation of findings

Many interviewees reported that their pain was not effectively controlled and had negatively impacted their life. Almost all used pharmacological approaches to manage their pain with very few using non-pharmacological interventions. The benefit of opioids and gabapentinoids in the management of chronic pain in elderly patients is limited and non-pharmacological approaches are undoubtedly preferable, with far less potential for patient harm.

Predominantly pharmacological chronic pain management was also reported in multiple publications. For example, a survey of urban and rural areas of North Dakota showed that 58% of respondents had chronic pain, with arthritis as the leading cause, and used oral medicine to control it. Those with chronic pain had significantly lower quality of life. Those residing in rural areas had significantly higher rates of chronic pain reported. Although not statistically significant, respondents from rural areas were less likely to use non-pharmacological approaches such as chiropractor, physical therapy, and massage [23]. Although these results were published in 2002, similar findings were highlighted in the current study suggesting that they are still relevant.

Use of non-pharmacological treatments was limited amongst those interviewed although they are considered a valid treatment option by national and international guidelines. A descriptive, comparative, cross-sectional pilot study of older adults in assisted and supported-living facilities who had chronic, non-cancer pain and who were taking pain medicines, reported that non-pharmacological interventions such as exercise and heat therapy significantly impacted chronic pain and perceived comfort scores [24]. Similarly, a recent systematic review evaluated the effectiveness, suitability, and sustainability of non-pharmacological pain management interventions for community-dwelling older adults. It reported that non-pharmacological methods of managing pain such as acupressure and acupuncture were effective in lowering pain levels in this population by helping to reduce the pain intensities of the participants. Thus, these can be promoted widely in the community [25].

Despite efforts by the Scottish Government and the different health authorities to improve access to care especially in remote and rural locations, interviewees in this study reported difficulties in accessing treatment options, both pharmacological and non-pharmacological approaches, where they resided, with some having to travel long distances for their appointments. This proved difficult for many who can no longer drive and was further complicated by the limited public transport available for them to use. Similar observations were noted in a recent systematic review by Suntai, Won, and Noh [26] which identified transportation-related issues as a major access barrier to pain care amongst rural older adults. These included lack of public transportation, lack of reliable drivers, and remoteness to the closest pain service providers.

To improve access to pain management, NHS Highland is currently redesigning its chronic pain service making sure the most complex cases come into the service and those less complex can be managed closer to home by GP practice staff. To enable this to happen, the chronic pain service is upskilling local primary care staff as well as offering self-management advice for patients in the form of peer support, websites and informative webinars. In part, it is hoped this will ameliorate the underuse of non-medication solutions (and lack of knowledge of these) and address the issues of travel that were highlighted in this study.

This study provides data that the chronic pain service needs more investment to fill the management gap which currently exists—alleviating pressure on GPs and generalist primary care services that often do not have either the time or the skills to deliver holistic pain management (as often behaviour change can take a number of sessions/appointments and considerable time to educate and reinforce beliefs/mood that does not lend itself well to 10-min appointments). Services with multidisciplinary teams that have the right time allocation and skills are needed across Highland and Scotland. However, these plans along with many other facilities offering pharmacological and non-pharmacological pain management approaches were put on hold or closed across the country due to the COVID-19 pandemic. This in turn meant that elderly adults are suffering from often extreme pain with no relief for prolonged periods of time.

### Further research

Future research needs to consider a larger geographical pool to determine whether the same views are shared by more patients across Scotland. There is also a need to develop solutions to overcome the barriers highlighted by participants especially in relation to access to pain management information and services. Additionally, there is a need to focus future research around understanding causes of pain and the attitudes to prescribing and resources available in primary care for the management of chronic pain considering the growing number of older adults most likely to present with chronic pain.

## Conclusion

Chronic pain and its management remain a significant issue among older adults residing in remote and rural areas that were interviewed. The main challenge to effective pain control was perceived to be access to chronic pain management services. Thus, future research should aim to explore this issue further.
